# Laying the Groundwork for Health: Eating Behaviour and Physical Activity in Preschoolers in Split-Dalmatia County, Croatia

**DOI:** 10.3390/children12060699

**Published:** 2025-05-29

**Authors:** Dora Bučan Nenadić, Lucija Štrkalj, Klara Zloić, Antonela Matana, Marija Selak, Matea Smoljo, Antonia Vlašić, Vanessa Ivana Peričić, Ela Kolak Gaurina

**Affiliations:** 1 Nutrition and Dietetics Department, University Hospital of Split, Spinčićeva 1, 21000 Split, Croatia; marija.selak@kbsplit.hr (M.S.); masmoljo@kbsplit.hr (M.S.); ekolak@kbsplit.hr (E.K.G.); 2 Croatian Association of Nutritionists, Ilica 134, 10000 Zagreb, Croatia; lucijastrkalj1@gmail.com (L.Š.); klara_0505@yahoo.com (K.Z.); antoniavlasic1509@gmail.com (A.V.); vanessa.ivana55@gmail.com (V.I.P.); 3 The University Department of Health Studies, University of Split, Ruđera Boškovića 35, 21000 Split, Croatia; antonela.matana@gmail.com

**Keywords:** children’s nutrition, children’s eating behaviour, sleep pattern, physical activity, eating habits

## Abstract

**Background/Objectives:** Overweight children and childhood obesity are growing public health concerns influenced by early-life nutrition and lifestyle. Irregular eating patterns, sedentary behaviour, and maladaptive eating behaviours significantly contribute to excess weight gain in children. This cross-sectional study comprehensively assessed physical activity, sleep, anthropometric parameters, and eating behaviours in preschool children in Split, Croatia, examining associations between eating behaviours and nutritional status indicators. **Methods:** A total of 429 children aged 4 to 7 years were recruited from kindergartens in Split-Dalmatia County. Parents completed a lifestyle questionnaire and the Children’s Eating Behaviour Questionnaire (CEBQ). Anthropometric measurements (weight, height, middle upper arm circumference, waist circumference) were recorded and BMI-for-age z-scores calculated. Physical activity and sleep patterns were assessed based on parental reports. **Results:** A total of 66% of the children had a healthy body weight, 12.6% were underweight, and 21.4% were overweight or obese. Significant sex differences were found in the CEBQ subscale “Slowness in Eating” (*p* = 0.04). Overweight or obese children showed a higher food responsiveness, while underweight/normal-weight children had greater emotional undereating and slowness in eating. No significant sex differences were observed regarding physical activity. Girls exhibited significantly more frequent daytime napping than boys. **Conclusions:** This study shows a significant prevalence of overweight and obese preschool children in Split, Croatia. The results underline the importance of promoting healthy eating behaviours and physical activity from an early age. This is the first study applying CEBQ in the Croatian population and suggests that the interventions should target diet quality and unfavourable eating behaviours to prevent future health risks.

## 1. Introduction

In recent decades, obesogenic modern environments have led to a significant rise in weight in the average population. Based on current trends, the World Federation predicts that 37% of the adult Croatian population will be living with obesity by 2035 [1]. These projections highlight a significant increase in the current levels of the issue and emphasize the importance of adopting and implementing a national action plan for obesity prevention. Childhood obesity is particularly on the rise. The annual growth rate of childhood obesity from 2020 to 2035 is expected to be 4.8%, compared to the adult obesity rate, which will be 2.0% [1].

A high-quality diet for preschool children that is aligned with nutritional recommendations [2,3] can have a positive impact on growth, reduce the risk of developing excess body fat, and have a long-term impact on the children’s health as adults. It has also been reported that unhealthy habits in childhood can lead to negative health consequences in adult life. For example, a study from Western Australia showed that poor dietary patterns in adolescence are unlikely to improve into early adulthood, particularly in male adolescents [4]. It was also reported that a higher body mass index (BMI) was positively associated with lower scores for healthy dietary patterns. Similarly, a Finnish prospective cohort study with a 21-year follow-up found that the food intake patterns established in childhood or adolescence were likely to be maintained in adulthood, and there was an association between male participants, smokers, and a lower consumption of fruit and vegetables [5]. However, a comprehensive review on dietary habits in childhood and adolescence and their correlation with cardiometabolic health concluded that studies focusing on children’s eating behaviours and risk factors for non-communicable diseases are needed [6].

Eating behaviour is closely related to energy intake, body mass index, and long-term weight gain, making it crucial to address and modify children’s food-related behaviours as a fundamental element of weight management [7,8]. Overweight and obese children are more likely to consume larger amounts of food when experiencing negative emotions, often engaging in overeating, exhibiting a greater desire for food, and being sensitive to food while experiencing a reduced sense of fullness [9,10,11,12,13]. In addition, a child’s eating behaviour may be related to the variety of their diet. Children who had a more diverse diet displayed reduced pickiness and a heightened overall interest in food [14]. Healthy dietary choices are important for the overall wellness and health outcomes in children. A systematic review has concluded that physical activity and healthy diet are correlated with better cognitive performance in children under 5 years of age [15]. Health-related quality of life was shown to be associated with higher levels of physical activity and lower levels of sedentary behaviour in primary school-aged children in Finland [16].

When it comes to the quality of nutrition and its health benefits for the individual, the Mediterranean diet (MeDi) is often recommended as the first choice [17]. Studies have shown that the MeDi offers numerous health benefits for children, such as a reduced risk of developing cardiovascular diseases [6,18], metabolic syndrome, and obesity, as well as regulated sleeping patterns and increased physical activity [19,20,21,22,23]. An earlier study conducted by the Croatian Association of Nutritionists on 598 preschool children found that 21% of preschool children in the Dalmatia region were overweight, with only 14% adhering to the principles of the MeDi [24]. A recently published study on nutritional habits of 6-to-10-year-old children in Croatia found a low intake of fruit and vegetables, and a higher intake of snacks and sweets [25]. Unfortunately, the trend of not meeting the recommendations for a healthy lifestyle is reported for physical activity as well. Only 53.8% of preschool children in Croatia could be described as “highly active” [26]. Moreover, sedentary behaviour and excessive screen time seem to be prevalent—it was found that three out of four preschoolers spent more than 2 h per day in front of a screen [27].

Given these discouraging results, the aim of this study was to explore the overall eating behaviours in preschool children, with a focus on how these behaviours relate to factors such as anthropometric measurements, sleep and physical activity habits, and adherence to the MeDi.

## 2. Materials and Methods

### 2.1. Study Design and Population

This cross-sectional study was conducted as part of a series of lectures and educational workshops organized by the Croatian Association of Nutritionists to promote healthy eating habits in kindergarteners and preschool-aged children. The study took place between September and December 2022 in randomly selected kindergartens in Split, Split-Dalmatia County, Croatia. For this study, it was determined that a minimum of 308 participants were needed for statistical models to be applicable and significant. The medium effect size (d = 0.5) was used for the comparison of two independent variables (sex) with the level of significance of 0.05 and the confidence level of 0.95, for which at least 210 participants were needed. Next, the medium effect size (d = 0.5) was used for the comparison of four independent variables (z-score—underweight, normal, overweight, and obese) with the level of significance of 0.05 and the confidence level of 0.95, for which at least 280 participants were required. For regression analysis with the confidence level of 0.95, it was determined that at least 105 participants were needed. Overall, at least 308 participants were needed for this study when drop-out bias (an extra 10% of participants) is considered as well. G* power statistical index 3.1.9.4. was applied.

After calculating the sample size, the following inclusion criteria were applied: The child had to be between 4 and 7 years old, attend an eight- or ten-hour program in a kindergarten where he or she was provided with four meals (two main meals and two snacks) by the facility, and only one child from the household could be included. Before participating in the study, verbal and written informed consent was obtained from the parents of each child and from the kindergarten management. Children who met any of the following criteria were excluded from the study: (1) refusal to participate, (2) presence of mental disorders that hindered participation in regular activities, (3) presence of nutritional allergies, (4) lack of parental consent, or (5) incomplete or unreturned questionnaires from the parents. Taking these criteria into consideration, this study included a total of 429 preschool children aged 4 to 7 years old.

### 2.2. Lifestyle Questionnaire

Parents who consented to their child’s participation in the study were asked to complete a lifestyle questionnaire designed to collect information on the characteristics of the population. The questionnaire covered various aspects, including demographic information such as birth weight and length as well as number of meals.

### 2.3. Children’s Eating Behaviour Questionnaire (CEBQ)

Eating behaviours were measured by using the Children’s Eating Behaviour Questionnaire (CEBQ). The CEBQ is a validated questionnaire consisting of 35 items, which are categorized into eight eating behaviours [25]. The questionnaire assesses both the food approach and food avoidance dimensions of a child’s eating behaviour. Ratings are provided on a 5-point Likert scale, ranging from “Never” (1) to “Always” (5). Seven questions were reverse scored. Food approach behaviours encompass food responsiveness—5 items, emotional overeating—4 items, enjoyment of food—4 items, and desire to drink—3 items. In addition, food avoidance behaviours include satiety responsiveness—5 items, slowness in eating—4 items, emotional undereating—4 items, and food fussiness—6 items [28]. In both cases, higher scores indicate a higher level of the corresponding eating style.

The reliability of the CEBQ was determined by applying Cronbach’s alpha statistical index. Overall reliability was satisfactory (α = 0.686). The Cronbach’s alpha was determined for each of the CEBQ scales: food responsiveness—0.748, emotional overeating—0.725, enjoyment of food—0.886, desire to drink—0.680, satiety responsiveness—0.655, slowness in eating—0.732, emotional undereating—0.739, and food fussiness—0.601. The validity of the CEBQ has been shown in previously published scientific papers [9,29]; however, as previously noted, this work is the first published report on the use of the CEBQ in the Croatian population. The authors can confirm that the translation has been done by a user proficient in the English language.

### 2.4. Physical Activity and Sleep Behaviour Questionnaire

The children’s parents answered questions about their child’s average daily activities, including sitting, walking, active play, type of physical activity (i.e., organized sports or exercise), and how many times per week and how long their child participates in said physical activity.

Sleep behaviour was assessed using questions about the duration of the child’s nighttime sleep and the frequency and duration of naps (if relevant).

### 2.5. The Mediterranean Diet Quality Index (KIDMED)

In addition to the study participants, parents were also requested to complete the KIDMED index questionnaire. This index was designed to assess adherence to the MeDi in children and young people aged two (2) to twenty-four (24) years. It is based on principles that reflect both Mediterranean eating habits and those that contradict them [8]. The index comprises sixteen questions, each of which can be answered with “yes” or “no”. Questions with negative connotations were assigned a value of −1, while those with positive connotations were given a value of +1 [24]. The total sum of all the values ranges from zero (0) to twelve (12). Based on this total, the results are classified into three levels:

1. >8, which indicates adherence to the optimal MeDi;
2. 4–7, suggesting that adjustments are needed to improve food intake according to the MeDi principles;
3. ≤3, representing a very low quality of nutrition according to the MeDi [30].

For determining the reliability of the KIDMED index questionnaire, Cronbach’s alpha statistical index was applied, and it was determined as satisfactory (α = 0.693). The validity of the KIDMED index questionnaire has been established in previous studies [23,31], as well as its application in the Croatian population [24].

### 2.6. Anthropometric Measurements

Anthropometric measurements were taken for each child in kindergarten, who was wearing light clothing. In order to provide the children with a pleasant and less anxiety-inducing experience, the anthropometric measurements were carried out in a playful and child-friendly way. It is important to emphasize that no child was forced or coerced to participate in the anthropometric measurement if they were not willing to do so, regardless of parental consent. Height was measured with a stadiometer, and weight with a calibrated Omron BF511 diagnostic scale (Omron, Kyoto, Japan) with an accuracy of one tenth of a decimal place [32]. In addition, the mid-upper arm circumference (MUAC) and waist circumference (WC) were measured using a non-stretchable, flexible tape measure. A BMI-to-age z-score was calculated for each as this particular classification system is endorsed by the World Health Organization (WHO) as it can accurately reflect nutritional status even at the extreme ends of the distribution. In addition, it allows the derivation of summary statistics, such as means and standard deviations of z-scores [33]. The z-scores were calculated based on the exact age in days according to the WHO standards and in months according to the 2007 WHO reference [32]. This comprehensive approach ensures an accurate assessment of each child’s BMI in relation to their age and allows a more accurate assessment of their nutritional status.

### 2.7. Statistical Analysis

Categorical data are represented by absolute and relative frequencies. Differences of categorical variables were tested by the chi-square test and, if necessary, by Fisher’s exact test. The normality of the distribution of numerical variables was tested by the Shapiro–Wilk test. Numerical data were described by the median and the limits of the interquartile range. Differences between the two independent groups were tested by Mann–Whitney’s U test. Differences in numerical variables in cases of 3 and more groups were tested by the Kruskal–Wallis test. The correlation between numeric variables was evaluated by Spearman’s correlation coefficient ρ (rho) and by the point-biserial correlation coefficient. All *p* values were two-sided. The significance level was set to Alpha = 0.05. MedCalc^®^ Statistical Software version 22.016 was used for statistical analysis [34]. To calculate the BMI-to-age z-score, the WHO software AnthroPlus 1.0.3. was used [35].

## 3. Results

Of the 429 children with a mean age of 5 years included in this study, 208 (48.5%) were girls. According to the BMI-to-age z-score values, 283 (66%) children had a healthy body weight, with 54 (12.6%) children classified as underweight and 92 (21.4%) as overweight or obese. The baseline characteristics and anthropometric measures of the study population are shown in Table 1.

Of the 429 children included, 65.5% spend an hour or more each day sitting or lying down. A total of 76.7% of the children reported playing actively for an hour or more, while 53.8% of the children participated in sports activities with an average duration of 60 min. In addition, 70.1% of parents reported that their children combined physical activity and sitting. No statistically significant difference was found between sexes in the aforementioned parameters. As for sleeping behaviour, girls napped significantly more often during the day (*p* = 0.009) than boys. Data about physical activity and sleeping behaviour are shown in Table 2.

When comparing the eating behaviour of boys and girls using the CEBQ, consisting of 8 subscales, a statistically significant difference was found for the subscale “Slowness in eating” (*p* = 0.04) (Table 3). When looking at the individual items of the CEBQ, a significant difference was found in relation to sex for the items “My child eats slowly” (*p* < 0.001) and “My child has a big appetite” (*p* = 0.02) (Supplementary Table S1).

The significant differences in certain CEBQ responses based on z-scores are shown in Table 4. Overweight and obese children reported eating more, as evidenced by the statements “If allowed, my child would eat too much” and “If I had a choice, my child would eat most of the time”. Children with underweight and normal BMIs reported eating more when they were happy, in contrast to overweight and obese children, as evidenced by the emotional undereating subscale. Slowness in eating was more commonly reported among children with underweight and normal BMIs. Furthermore, children with obesity exhibited the lowest levels of food fussiness, as evidenced by a reduced tendency to reject foods without prior tasting. Satiety responsiveness was inversely associated with higher BMI categories; overweight and obese children were less likely to exhibit signs of fullness, as indicated by higher agreement with the item “My child has a big appetite”. In contrast, children with underweight or normal BMIs were more frequently described as becoming full easily and were less inclined to consume meals immediately after a snack. The non-significant results are shown in Supplementary Table S2.

Predictive factors for being underweight, overweight, and obese are expressed as odds ratios (ORs) and can be found in Table 5. The only significant predictive factor for underweight children was emotional undereating, with an OR of 1.47. The food responsiveness OR was low in overweight children (0.61). Interestingly, a high OR for slowness in eating was reported in overweight children, while it was low in obese children (1.33 and 0.41, respectively). The OR for enjoyment of food was extraordinarily high in obese children (1.94). There were no statistically significant differences in emotional overeating, desire to drink, food fussiness, or satiety responsiveness in any of the groups.

**Figure 1:**
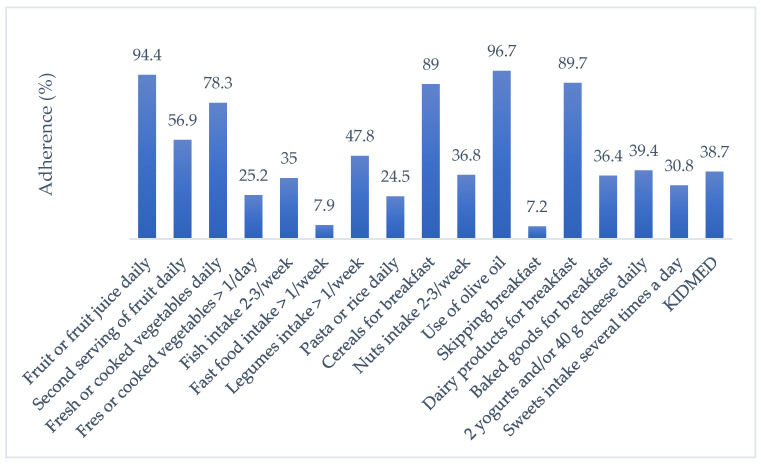
The overall adherence to the KIDMED score and its components.

Regarding adherence to the principles of the MeDi, 38.7% of the study population had high MeDi compliance; 55.9% had moderate MeDi compliance; and 5.4% of participants had low MeDi compliance. The highest compliance with the KIDMED components was found in terms of distributed scores for olive oil use, daily fruit and fruit juice consumption, and dairy consumption for breakfast. The overall adherence to the KIDMED score and its components are shown in Figure 1.

**Figure 2:**
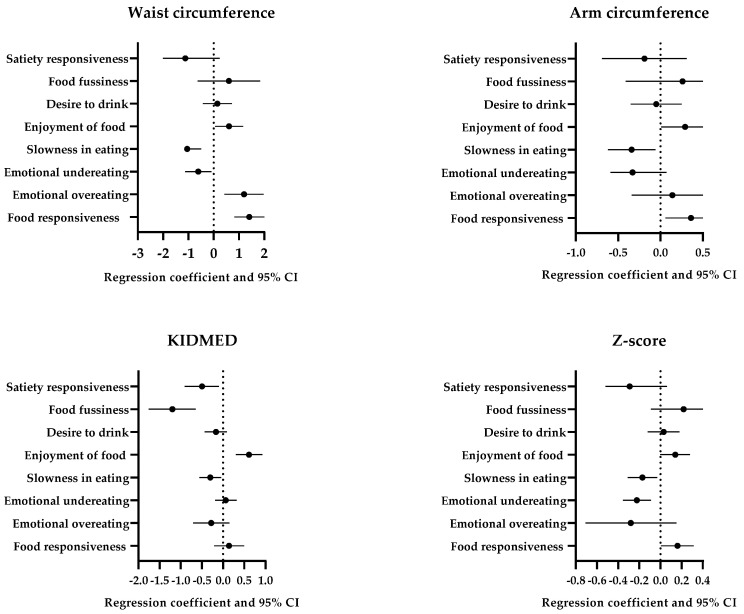
Association between CEBQ, anthropometric parameters, and KIDMED index.

For the food approach scales, following adjustment for confounders, higher food responsiveness was associated with higher arm (*p* = 0.02) and waist (*p* < 0.001) circumferences and z-score (*p* = 0.04, Figure 2); higher emotional overeating was associated with higher waist circumference (*p* = 0.002); and higher enjoyment of food was associated with higher waist circumference (*p* = 0.03) and KIDMED index (*p* < 0.001). For the food avoidance scales, greater slowness in eating was associated with arm (*p* = 0.02) and waist (*p* < 0.001) circumferences, z-score (*p* = 0.03), and KIDMED index (*p* = 0.03); greater emotional undereating was associated with arm and waist circumference (*p* = 0.02) and z-score (*p* = 0.002); and greater satiety responsiveness was associated with waist circumference and z-score (*p* = 0.01) as well as KIDMED index (*p* = 0.02). Desire to drink was not associated with any measures of body composition or KIDMED index.

## 4. Discussion

Of the 429 preschool children included in the present study, 21.5% were found to be overweight and obese, with no significant differences in age or gender. These findings are consistent with data from the fifth round of the Childhood Obesity Surveillance Initiative (COSI), in which 29% of children aged 7–9 years were overweight or obese [36]. Although these results are devastating, they were to some extent expected, considering that obesity is on the rise across the European region, particularly in Croatia with the prevalence of overweight people among women and men being 58% and 73%, respectively [37]. An overweight and obese paediatric population remains one of the greatest public health challenges in the WHO European Region, as it significantly affects children’s immediate physical and mental health, educational attainment, and quality of life and increases the risk of obesity and noncommunicable diseases later in life [36].

Children are recommended to engage in at least 180 min of physical activity per day [38], with 120 min at a moderate to vigorous intensity as recommended for children aged 2 to 3 years and older in the United States [39]. However, these targets are often not met. In Japan, only 34–35% of 3-to-4-year-olds met the guidelines [40], and in Spain, although all children were active for 60 min a day, only 50% reached 120 min [41]. In the present work, only 49.4% of children played actively for more than an hour a day, with 70.1% doing a combination of sitting and activity and only 25% being very active. No significant sex differences were found, which is consistent with the Canadian results [42] although moderate to vigorous activity was reported in 3-year-old boys in a Finnish study [43]. Promoting physical activity, especially in kindergartens and schools, is crucial as activity levels are higher during school hours than outside of school [41].

A systematic review with a total number of 52,977 children subjects and 55,478 adolescent found that adherence to the MeDi was directly associated with physical activity and inversely associated with sedentary behaviour, while the results for sex, age, socioeconomic status, and weight status were not consistent, similar to the results shown here [23]. From the above, it can be concluded that children with appropriate eating behaviours generally lead healthier lifestyles, either due to better parental feeding behaviours and monitoring [44] or due to the abundance of nutrients that help maintain energy expenditure during physical activity [45]. Overall, physical activity is a crucial part of children’s wellbeing and development, and should be encouraged, particularly since most of the studies show a lack of physical activity. In addition, both healthy dietary patterns and physical activity are key to children’s health, and their interplay should be further explored.

Our study found a significant difference in nap times between boys and girls (*p* = 0.009), with girls napping more. Research results on sex differences in nap times are inconsistent: a Chinese study (9–11 years) also found that girls napped more [46], while a New Zealand study (3–7 years) found no difference [47]. A meta-analysis showed high variability in napping behaviour in 3–5-year-olds and found that genetic factors influence napping behaviour most strongly in 0–2-year-olds, while environmental factors dominate after the second year of life [48]. Therefore, kindergartens play a key role in establishing healthy napping habits.

There is increasing evidence that sleep influences eating behaviour. The quantity and quality of sleep can influence energy intake not only through an increase in the number of meals consumed during the day, frequency of snacking, and preference for energy-dense foods, but also through psychological stress and changes in appetite hormones [49]. The majority of the above studies were conducted with children and adolescents older than those included in the current study, who, according to the literature review, represent the youngest population in which sleep has been associated with diet quality [50,51]. Therefore, this area may be of interest for future research.

Eating behaviour in children can be described using the eight CEBQ subscales: food responsiveness, emotional undereating, emotional overeating, satiety responsiveness, food fussiness, desire to drink, slowness in eating, and enjoyment of food. The CEBQ has been widely used in scientific studies, but to the best of our knowledge, it has not been included in research regarding dietary habits of Croatian children. Therefore, this is the first study reporting on the insights of the CEBQ application in the population of Croatian kindergarteners. In the present study, girls showed higher scores for the CEBQ component “Slowness in eating”. Interestingly, the opposite was found in a recent study of 197 preschool children [52]. No significant sex differences in eating behaviour were found in the previous studies, except in two studies where differences were found in the “Desire to drink” scale with the higher scores in boys compared to girls [53]. As discussed in the aforementioned studies, it is important to determine at what age gender differences in diet behaviour become apparent. Adolescence is a period that is particularly emphasized as girls become more aware of their body image [52]. Diverse results were observed regarding the impact of age on children’s eating behaviour. The STEPS study found that the food approach dimension and all subscale variables were higher at 2 years of age compared with 5 years of age [54]. Contrary to that, Wardle et al. found that “Food responsiveness“ and “Enjoyment of food“ increased as children grew older, whereas “Satiety responsiveness“ and “Slowness in eating“ both decreased with age [28]. In contrast, Farrow and Blissett observed a similarity in eating behaviour between children aged 2 and 5 years [55]. Interestingly, both previously mentioned studies indicated a decrease in the desire to drink as children age.

Children with excessive body mass were found to have a more pronounced interest in food, as they scored high on the “Food responsiveness” scale. On the other hand, malnourished children had lower scores on the “Emotional undereating” and “Satiety responsiveness”, which may indicate a lack of interest in food [56,57]. Emotional undereating was a significant predictive factor for underweight children, and it was also reported that their food intake is higher when experiencing happiness. Similarly, a study on 4-year-olds in the Netherlands also found a positive association between lower BMIs and emotional undereating [57]. On the other hand, a UK-based study found no significant differences in emotional undereating among children aged 7 to 12 across different BMI categories [12]. Also, Buja et al. found that good adherence to the MeDi was associated with a lower risk of “Emotional undereating”, while no such association emerged for “Emotional overeating” [58].

As our study and other studies from the Netherlands, the United Kingdom, Brazil, and Ireland show, food intake is often more pronounced in children with high BMIs; i.e., they are more responsive to external cues than to hunger [56,59,60]. Surprisingly, responsiveness to food was a lower predictor of being overweight (OR = 0.61), possibly due to factors such as food insecurity and the child’s temperament, both of which are associated with increased responsiveness [61,62]. Although food responsiveness alone is not a strong predictor of weight, it is important because of its association with later eating disorders [60]. Satiety responsiveness, the ability to regulate food intake based on feelings of fullness, was lower in overweight and obese children, which is consistent with findings in toddlers [59], but differs from a Chinese study on older children [63]. Biological differences in appetite hormones were also observed [64], highlighting the need to understand satiety to prevent obesity. Food fussiness was the lowest in obese children, and enjoyment of food strongly predicted obesity (OR = 1.94, *p* = 0.04), which is consistent with previous studies [12]. Even though these findings may be somewhat expected, the link between a child’s weight and food fussiness is complicated. A systematic review has concluded that there was no association between food neophobia and weight, and there were no clear results for picky eating and weight in childhood [65]. Nevertheless, decreasing food fussiness in children is important for improving the overall nutrition of their diet [66].

Slowness in eating was more common in children with an underweight or normal BMI. It predicted a lower risk of obesity, but interestingly was a positive predictor of being overweight. Since slow eating supports satiety and healthy weight regulation [67], early promotion of mindful eating, especially in kindergarten, is essential. In the present study, slowness in eating was positively correlated with reports of children leaving their food on the plate at the end of a meal and children being satiated more easily (Supplementary Table S3). Research shows that eating more slowly gives internal signals of fullness time to register, which can help children better self-regulate their food intake [68]. Therefore, slowness in eating may protect against the development of disordered eating patterns and obesity later in life [60].

More notably, parents’ eating habits have a significant influence on children’s eating behaviour, regardless of demographic factors such as gender, age, or socioeconomic status. Although the exact mechanisms are still unclear, elements such as the home and social environments play a crucial role in shaping children’s dietary beliefs and behaviour [57]. As primary role models, parents shape the home eating environment and influence children’s food preferences and eating habits, thus making a decisive contribution to the development of their eating behaviour [69]. However, certain eating practices can have unintended consequences. The use of food to control a child’s emotions or behaviour in infancy has been associated with increased appetite from infancy to early childhood, regardless of initial appetite characteristics [70]. Similarly, the use of food as a reward or condition promotes the tendency to emotional overeating, which may also be a response to pre-existing emotional overeating in children [71]. This highlights the significant influence of the parental role in shaping children’s long-term dietary behaviour and their relationship with food and thus represents an area that requires further investigation in order to develop comprehensive interventions.

The present study, which emerged from the second phase of the Croatian Association of Nutritionists’ project entitled “Growing Healthy”, showed a high adherence to the MeDi principles of around 39 percent. On the other hand, only 14% of 598 participants showed high MeDi adherence in the first stage of the before-mentioned project [24]. Considering that both phases of the project involved children of the same age and from the same geographical area and who received three to four meals in kindergarten, the resulting difference could reflect the quality of school-based feeding. In this case, the impact of the coronavirus pandemic on awareness of proper nutrition and body composition should not be overlooked. Adherence to the MeDi and the implementation of the KIDMED index has been included in other studies regarding children and adolescents in Croatia. Obradović Salcin et al. have found high compliance to the MeDi in 5- and 6-year-old children in Split County, Croatia, with girls having higher adherence than boys [72]. Sila et al. found that the KIDMED index was higher for participants (mean age = 9.96 years) in the Mediterranean region as opposed to the continental region (5.57 and 4.91, respectively), but overall the MeDi adherence was low (15.8% of participants had good compliance to the MeDi) [73]. Similarly, low practice of the MeDi was reported in kindergarteners in the Mediterranean region of Croatia, where 23.7% of 2- to 7-year-old children had good MeDi adherence [74].

A similar trend was observed in other Mediterranean countries [23,75]. A systematic review article that examined the dietary intake of preschool children, including the multinational IDEFICS (Identification and prevention of dietary- and lifestyle-induced health effects in children and infants) study and several national studies from Portugal, Spain, Italy, Greece, and France, found that most children in the observed countries had low adherence to the principles of the MeDi [75]. This adherence was associated with an increased risk of being overweight or obesity [76]. The MeDi is considered one of the healthiest dietary patterns with numerous positive effects on the body composition and general health of the individual [75]. It is therefore surprising that Mediterranean countries such as Cyprus, Greece, and Italy are at the forefront of rates of overweight and obese children and adults [75]. There are several possible explanations for this paradox, the most significant of which is the increasingly sedentary lifestyle in all age groups [74] and the deviation from the traditional MeDi along with the adoption of a Western dietary pattern characterized by an increased intake of processed foods, sugar, and saturated fats [77,78]. In terms of children’s body weight status, anthropometric parameters, MeDi adherence, and their responses to the CEBQ, the present findings are consistent with the results of previous studies [28,29,73]. All of the above points to the departure from the traditional dietary patterns and emphasises the need for nutritional intervention in this particular population of participants.

This study has several limitations. Its cross-sectional design does not allow conclusions about causality. The data were based on self-reporting by parents, which may lead to recall bias, social desirability bias, and subjective interpretations of behaviour. Moreover, the dietary patterns and lifestyle of the parents were not assessed. The questionnaires used also provide a brief overview of dietary habits and eating behaviours, but may not capture the full complexity of the children’s diets or the context of their eating environment. The regional focus on the Split-Dalmatia County limits the possibility of generalization to other population groups or regions with different socioeconomic, cultural, or environmental contexts. Finally, selection bias is possible because only children attending kindergarten and children whose parents provided consent were included.

Based on these limitations, several avenues for future research are recommended. Including lifestyle and dietary assessments for parents would offer a more comprehensive understanding of the home and kindergarten environments influencing children’s nutrition. Additionally, incorporating a more detailed foods list—particularly with categories for foods recommended in limited amounts (e.g., sugary beverages and sweets)—could yield more nuanced insights together with standardised questionnaires such as the CEBQ and the KIDMED. Finally, this study employed a cross-sectional design, which, while informative, does not allow for the assessment of dietary and behavioural changes over time. Future research would benefit from a longitudinal design that follows children through kindergarten, preschool, and potentially into early school years. Such an approach would provide a richer understanding of the development and evolution of dietary habits and their impact on long-term health.

Practically, interventions to prevent childhood obesity can be divided into four categories: nutritional, physical activity-based, behaviour change-based, and multicomponent [79]. Their effectiveness varies depending on the approach and age group [80]. For children aged 0–5 years, physical activity interventions show moderate effectiveness, but there is insufficient data on the effectiveness of diet-only interventions, potential adverse effects, or long-term outcomes as most studies follow participants for 12 months or less [81,82]. A pilot study focused on children aged 2–15 years included a weekly gardening session, a 7-week cooking and nutrition workshop, and social events for parents and children and showed promising results for addressing childhood obesity and healthier dietary choices [83]. Therefore, a more comprehensive approach might be productive in this endeavour.

## 5. Conclusions

The findings of this study, conducted among preschool-aged children, revealed a concerning prevalence of overweight and obese children, accompanied by low levels of physical activity and moderate adherence to the Mediterranean Diet (MeDi) principles. Notably, overweight and obese children demonstrated significantly higher food responsiveness, along with lower satiety responsiveness and reduced food fussiness, compared to their underweight and normal-weight peers. In contrast, children of normal or underweight status exhibited greater emotional eating when happy and a tendency toward slower eating behaviours.

These results underscore the preschool period as a critical developmental window for the establishment of eating behaviour patterns and the implementation of effective obesity prevention strategies. In light of the high prevalence of obesity observed, there is an urgent need for comprehensive nutritional interventions in Croatia. This includes the formulation of updated, evidence-based national dietary guidelines and the introduction of age-appropriate educational initiatives targeting both children and caregivers.

## Figures and Tables

**Table 1:** Baseline characteristics and anthropometric measures of study population.

	F *n* = 208 (48.5%)	M *n* = 221 (51.5)	Total *n* = 429	p *
Age (*n*, %)				
<5 years	86 (41.3)	84 (38)	170 (39.6)	0.40
5 years	64 (30.8)	62 (28.1)	126 (29.4)	
≥6 years	58 (27.9)	75 (33.9)	133 (31)	
Age (median, IQR)	5 (4–6)	5 (4–6)	5 (4–6)	0.13
Height, m (median, IQR)	1.13 (1.1–1.19)	1.1 (1.1–1.2)	1.13 (1.06–1.2)	0.06
Birth length, cm (median, IQR)	50 (49–52)	51 (50–53)	51 (50–52)	<0.001
Weight, kg (median, IQR)	19.6 (16.9–22)	20 (17.9–23.5)	19.8 (17.4–22.8)	0.02
Birth weight, g (median, IQR)	3390 (3130–3730)	3510 (3180–3880)	3490 (3150–3800)	0.004
WC, cm (median, IQR)	51.9 (49.5–54.5)	53 (51–56)	52.5 (50–55)	<0.001
MUAC, cm (median, IQR)	16.5 (16–18)	17 (16–18)	16.7 (16–18)	0.22
Percentile (median, IQR)	56.9 (34.5–77.9)	61.2 (34.5–82.7)	59.2 (34.6–80.6)	0.49
BMI-to-age z-score (median, IQR)	0.2 (−0.4–0.8)	0.3 (−0.4–1)	0.2 (−0.4–0.9)	0.43
Main meals, *n* (median, IQR)	3 (3–3)	3 (3–3)	3 (3–3)	0.87
Snacks, *n* (median, IQR)	1 (1–2)	2 (2–2)	2 (2–2)	0.001
Household income				0.05
2850–5700	4 (2)	11 (5.1)	15 (3.6)	
5700–8600	28 (14)	21 (9.7)	49 (11.8)	
8600–11,400	33 (16.5)	52 (24.1)	85 (20.4)	
11,400<	135 (67.5)	132 (61.1)	267 (64.2)	

Abbreviations: F—female, M—male, *n*—number, IQR—interquartile range, WC—waist circumference, MUAC—mid-upper arm circumference. * *p*-values were obtained with chi-square test for categorical variables and Mann–Whitney U test for non-parametric numerical variables.

**Table 2:** Physical activity and sleeping behaviour and their components regarding sex.

	F *n* = 208 (48.5%)	M *n* = 221 (51.5%)	Total *n* = 429	*p* *
How much time per day does your child spend in the following activities?				
Sitting (watching TV, lying in bed or on the couch, playing while sitting—video games, toys, puzzles, colouring); *n*, %				
15 min	2 (1)	11 (5)	13 (3)	0.14
30 min	31 (14.9)	29 (13.1)	60 (14)	
45 min	38 (18.3)	37 (16.7)	75 (17.5)	
1 h	62 (29.8)	73 (33)	135 (31.5)	
>1 h	75 (36.1)	71 (32.1)	146 (34)	
Walking (a walk to school, a walk to the park and in the park, a walk in the shopping centre or to the store); *n*, %				
15 min	4 (1.9)	4 (1.8)	8 (1.9)	0.77
30 min	20 (9.6)	30 (13.6)	50 (11.7)	
45 min	26 (12.5)	27 (12.2)	53 (12.4)	
1 h	45 (21.6)	49 (22.2)	94 (21.9)	
>1 h	113 (54.3)	111 (50.2)	224 (52.2)	
Active playing (playing tag, riding a bicycle or scooter, playing with a ball); *n*, %				
15 min	6 (2.9)	6 (2.7)	12 (2.8)	0.28
30 min	28 (13.5)	20 (9)	48 (11.2)	
45 min	22 (10.6)	18 (8.1)	40 (9.3)	
1 h	60 (28.8)	57 (25.8)	117 (27.3)	
>1 h	92 (44.2)	120 (54.3)	212 (49.4)	
How many days a week does your child spend doing the following and similar activities: gymnastics, karate, football, swimming, or dancing?; median, IQR				
	2 (2–3) min 1–max 5 *n* = 112	3 (2–3) min 1–max 5 *n* = 116	3 (2–3) min 1–max 5 *n* = 228	0.06
How much time (in minutes) does your child spend doing the following and similar activities: gymnastics, karate, football, swimming, or dancing?; median, IQR				
	60 (50–60) min 30–max 120 *n* = 112	60 (60–60) min 30–max 120 *n* = 116	60 (60–60) min 30–max 120 *n* = 228	0.85
How many times a day does your child take a nap?; *n*, %				
never	76 (36.5)	107 (49.1)	186 (43.4)	0.009
once	131 (63)	111 (50.9)	242 (56.4)	
twice	1 (0.5)	0	1 (0.2)	
How long (in minutes) does your child take a nap?; median, IQR				
	60 (60–90) min 15–max 120 *n* = 132	60 (60–120) min 20–max 180 *n* = 111	60 (60–90) min 15–max 180 *n* = 243	0.95
How many hours does your child spend sleeping during the night?; median, IQR				
	10 (9–10) min 7–max 12	10 (9–10) min 1 max 12	10 (9–10) min 1 max 12	0.50
Which of the following phrases best describes your child’s activity at home?				
mostly sitting	11 (5.3)	10 (4.5)	21 (4.9)	0.09
combination of sitting and playing	154 (74.4)	146 (66.1)	300 (70.1)	
very active	42 (20.3)	65 (29.4)	107 (25)	

Abbreviations: F—female, M—male, *n*—number, IQR—interquartile range, min—minimum, max—maximum. * *p*-values were obtained with chi-square test and Fisher’s exact test for categorical variables and Mann–Whitney U test for non-parametric numerical variables.

**Table 3:** Children’s eating behaviour questionnaire and subscale results grouped by sex.

CEBQ Subscales	Median, IQR			p *
	F (*n* = 208)	M (*n* = 221)	Total (*n* = 429)	
Food responsiveness	2 (1.5–2.25)	2 (1.5–2.5)	2 (1.5–2.5)	0.73
Emotional overeating	1.5 (1–2)	1.3 (1–1.8)	1.25 (1–1.75)	0.79
Emotional undereating	2.5 (2–3)	2.5 (2–3.3)	2.5 (2–3)	0.87
Slowness in eating	3 (2.5–3.5)	2.8 (2.3–3.3)	3 (2.25–3.29)	0.04
Enjoyment of food	3.8 (3.3–4.3)	4 (3.3–4.3)	4 (3.3–4.3)	0.35
Desire to drink	2.7 (2.3–3.3)	2.7 (2.3–3.3)	2.7 (2.3–3.3)	0.16
Food fussiness	2.7 (2.6–3)	2.9 (2.6–3)	2.9 (2.6–3)	0.58
Satiety responsiveness	2.8 (2.6–3.2)	2.8 (2.6–3.2)	2.8 (2.6–3.2)	0.81

Abbreviations: F—female, M—male, IQR—interquartile range. * *p*-values were obtained with Mann–Whitney U test for non-parametric numerical variables.

**Table 4:** Children’s eating behaviour questionnaire and subscale results grouped by z-score.

	Subscale	Median (IQR)					p *
		Underweight BMI	Normal BMI	Overweight BMI	Obese BMI	Total	
If allowed, my child would eat too much.	FR	1 (1–2)	1 (1–2)	2 (1–3)	2 (1–3)	1 (1–2)	<0.001
Given the choice, my child would eat most of the time.	FR	1 (1–2)	1 (1–2)	2 (1–3)	1 (1–2)	1 (1–2)	0.04
My child eats more when s/he is happy.	EUE	3 (2–4)	3 (2–3)	2 (1–3)	2 (1–3)	3 (1–3)	<0.001
My child finishes their meal fast.	SE	3 (3–4)	3 (3–4)	3 (3–4)	3 (2–3)	3 (3–4)	0.02
My child takes over 30 min to finish a meal.	SE	3 (1–3)	2 (2–3)	2 (1–3)	2 (1–2)	2 (1–3)	0.02
As the meal goes on, my child eats more and more slowly.	SE	2 (2–3)	2 (2–3)	2 (1–3)	2 (1–3)	2 (2–3)	0.04
My child decides s/he doesn’t like a food without even trying it.	FF	3 (2–3)	3 (2–4)	3 (2–4)	2 (1–3)	3 (2–4)	0.03
My child has a big appetite.	SR	3 (2–4)	3 (2–4)	3 (3–4)	3 (3–4)	3 (2–4)	0.04
My child gets full easily.	SR	3 (3–4)	3 (3–3)	3 (2–3)	3 (2–3)	3 (2–3)	<0.001
My child cannot eat a meal immediately after a snack.	SR	3 (2–4)	3 (2–3)	2 (1–3)	2 (1–3)	3 (2–3)	0.03

For each statement, a corresponding subscale (2nd column) is indicated (only statistically significant values shown). Abbreviations: IQR—interquartile range, BMI—body mass index, FR—food responsiveness, EUE—emotional undereating, SE—slowness in eating, FF—food fussiness, SR—satiety responsiveness, p *—values were obtained with the Kruskal–Wallis test.

**Table 5:** Predictive factors for underweight, overweight, and obese body masses.

	Underweight		Overweight		Obese	
	OR (95% CI)	p *	OR (95% CI)	p *	OR (95% CI)	p *
Food responsiveness	1.03 (0.69–1.53)	0.89	0.61 (0.46–0.80)	<0.001	1.44 (0.86–2.41)	0.16
Emotional overeating	1.11 (0.67–1.84)	0.68	0.85 (0.59–1.21)	0.37	1.60 (0.82–3.13)	0.17
Emotional undereating	1.47 (1.03–2.08)	0.03	1.08 (0.85–1.38)	0.51	0.83 (0.50–1.38)	0.48
Slowness in eating	1.03 (0.71–1.49)	0.86	1.33 (1.02–1.73)	0.04	0.41 (0.22–0.74)	0.003
Enjoyment of food	0.99 (0.68–1.43)	0.95	0.84 (0.64–1.09)	0.18	1.94 (1.02–3.69)	0.04
Desire to drink	1.06 (0.73–1.53)	0.78	1.01 (0.78–1.32)	0.92	1.02 (0.59–1.78)	0.93
Food fussiness	0.51 (0.22–1.16)	0.11	1.29 (0.73–2.27)	0.38	0.57 (0.17–1.87)	0.35
Satiety responsiveness	1.50 (0.83–2.72)	0.18	1.22 (0.81–1.84)	0.34	0.54 (0.23–1.26)	0.16

Abbreviations: OR—Odds Ratio, p *—values were obtained with Mann–Whitney U test for non-parametric numerical variables.

## Data Availability

Raw data can be found from the corresponding author via e-mail: dorabucan@gmail.com; elakolak93@gmail.com.

## Supplementary Materials

The following supporting information can be downloaded at https://www.mdpi.com/article/10.3390/children12060699/s1, Table S1: Children’s eating behaviour questionnaire and subscale results grouped by sex; Table S2: Children’s eating behaviour questionnaire and subscale results grouped by z-score; Table S3: Spearman’s correlation indexes of slowness in eating and meal completion.

## Abbreviations

The following abbreviations are used in this manuscript:

MeDi	Mediterranean diet
KIDMED	Mediterranean Diet Quality Index for children
CEBQ	Children’s Eating Behaviour Questionnaire
BMI	Body mass index
MUAC	Middle upper arm circumference
WC	Waist circumference
WHO	World health organization
IQR	Interquartile range
M	Male
F	Female
n	Number
Min	Minimum
Max	Maximum
OR	Odds ratio
FR	Food responsiveness
EUE	Emotional undereating
SE	Slowness in eating
FF	Food fussiness
SR	Satiety responsiveness
