# Supplementary material for: Laying the Groundwork for Health: Eating Behaviour and Physical Activity in Preschoolers in Split-Dalmatia County, Croatia
Source: Children (Basel). 2025 May 29;12(6):699. doi: 10.3390/children12060699 (PMC12191604; doi:10.3390/children12060699)
Supplement: Supplementary file 1 [file children-12-00699-s001.zip › Bucan Nenadic_2025_Children_supplemetary_S1.pdf]

Supplementary materials

Table S1. Children's eating behaviour questionnaire and subscale results grouped by sex.

	Median, IQR			p*
	**F** (n = 208)	**M** (n = 221)	**Total** (n = 429)	
**Food responsiveness**	2 (1.5 - 2.25)	2 (1.5 - 2.5)	2 (1.5 - 2.5)	0.73
Even if my child is full up s/he finds room to eat his/her favourite food.	3 (2 - 3)	3 (2 - 3)	3 (2 - 3)	0.51
My child is always asking for food.	3 (3 - 4)	3 (3 - 4)	3 (3 - 4)	0.81
If allowed to, my child would eat too much.	1 (1 - 2)	1 (1 - 2)	1 (1 - 2)	0.28
Given the choice, my child would eat most of the time.	1 (1 - 2)	1 (1 - 2)	1 (1 - 2)	0.54
If given the chance, my child would always have food in his/her mouth.	1 (1 - 2)	1 (1 - 2)	1 (1 - 2)	<0.99
**Emotional overeating**	1.5 (1 - 2)	1.3 (1 - 1.8)	1.25 (1 - 1.75)	0.79
My child eats more when worried.	1 (1 - 2)	1 (1 - 2)	1 (1 - 2)	0.58
My child eats more when annoyed.	1 (1 - 2)	1 (1 - 2)	1 (1 - 2)	0.62
My child eats more when anxious.	1 (1 - 2)	1 (1 - 2)	1 (1 - 2)	0.90
My child eats more when s/he has nothing else to do.	1 (1 - 2)	1 (1 - 2)	1 (1 - 2)	0.82
**Emotional under eating**	2.5 (2 - 3)	2.5 (2 - 3.3)	2.5 (2 - 3)	0.87
My child eats less when angry.	3 (2 - 3)	2 (1 - 3)	3 (1 - 3)	0.23
My child eats less when s/he is tired.	3 (2 - 4)	3 (2 - 4)	3 (2 - 4)	0.80
My child eats more when she is happy.	3 (1.3 - 3)	3 (1 - 3)	3 (1 - 3)	0.28
My child eats less when upset.	2 (1 - 3)	2 (1 - 3)	2 (1 - 3)	0.84
**Slowness in eating**	3 (2.5 - 3.5)	2.8 (2.3 - 3.3)	3 (2.25 - 3.29)	0.04
My child finishes his/her meal quickly.	3 (3 - 4)	3 (2 - 4)	3 (3 - 4)	0.25
My child eats slowly.	4 (3 - 4)	3 (3 - 4)	4 (3 - 4)	<0.001
My child takes more than 30 minutes to finish a meal.	2 (2 - 3)	2 (1 - 3)	2 (1 - 3)	0.32

My child eats more and more slowly during the course of a meal.	2 (2 - 3)	2 (1 - 3)	2 (2 - 3)	0.62
**Enjoyment of food**	3.8 (3.3 - 4.3)	4 (3.3 - 4.3)	4 (3.3 - 4.3)	0.35
My child loves food.	4 (3 - 5)	4 (4 - 5)	4 (4 - 5)	0.09
My child is interested in food.	4 (3 - 4)	4 (3 - 4)	4 (3 - 4)	0.21
My child looks forward to mealtimes.	4 (3 - 4)	4 (3 - 4)	4 (3 - 4)	0.82
My child enjoys eating.	4 (3 - 4)	4 (3 - 4)	4 (3 - 4)	0.62
**Desire to drink**	2.7 (2.3 - 3.3)	2.7 (2.3 - 3.3)	2.7 (2.3 - 3.3)	0.16
My child is always asking for a drink.	4 (3.3 - 5)	4 (3 - 5)	4 (3 - 5)	0.61
If given the chance, my child would drink continuously throughout the day.	2 (1 - 2)	2 (1 - 3)	2 (1 - 3)	0.06
If given the chance, my child would always be having a drink.	2 (2 - 3)	2 (2 - 3)	2 (2 - 3)	0.11
**Food fussiness**	2.7 (2.6 - 3)	2.9 (2.6 - 3)	2.9 (2.6 - 3)	0.58
My child refuses new foods at first.	3 (2 - 4)	3 (3 - 4)	3 (2 - 4)	0.25
My child enjoys tasting new foods.	3 (2 - 3)	3 (2 - 3)	3 (2 - 3)	0.41
My child enjoys a wide variety of foods.	3 (2 - 4)	3 (2 - 4)	3 (2 - 4)	0.30
My child is difficult to please with meals.	2 (1 - 3)	2 (1 - 3)	2 (1 - 3)	0.24
My child is interested in tasting food s/he hasn't tasted before.	3 (2 - 4)	3 (2 - 4)	3 (2 - 4)	0.91
My child decides that s/he doesn't like a food, even without tasting it.	3 (2 - 4)	3 (2 - 4)	3 (2 - 4)	0.20
**Satiety responsiveness**	2.8 (2.6 - 3.2)	2.8 (2.6 - 3.2)	2.8 (2.6 - 3.2)	0.81
My child has a big appetite.	2 (2 - 4)	3 (3 - 4)	3 (2 - 4)	0.02
My child leaves food on his/her plate at the end of a meal.	3 (2 - 3)	3 (2 - 3)	3 (2 - 3)	0.21
My child gets full before his/her meal is finished.	3 (2 - 4)	3 (2 - 3)	3 (2 - 3)	0.36
My child gets filled up easily.	3 (2 – 3.8)	3 (2 - 3)	3 (2 - 3)	0.64

My child cannot eat a meal if s/he has had a snack just before.	3 (2 - 3)	2 (2 - 3)	3 (2 - 3)	0.46

Abbreviations: F – female, M – male, IQR – interquartile range. *p-values were obtained with Mann-Whitney U test for non-parametric numerical variables.
