# Supplementary material for: Laying the Groundwork for Health: Eating Behaviour and Physical Activity in Preschoolers in Split-Dalmatia County, Croatia
Source: Children (Basel). 2025 May 29;12(6):699. doi: 10.3390/children12060699 (PMC12191604; doi:10.3390/children12060699)
Supplement: Supplementary file 1 [file children-12-00699-s001.zip › Bucan Nenadic_2025_Children_supplemetary_S2.pdf]

Supplementary Table S2. Children's eating behavior questionnaire and subscales results grouped by z-score.

	Median (IQR)					*p**
	Underweight BMI	Normal BMI	Overweight BMI	Obese BMI	Total	
My child is constantly asking for food.	3 (3 - 4)	3 (3 - 4)	3 (3 - 4)	3 (3 - 4)	3 (3 - 4)	0,24
If allowed, my child would eat too much.	1 (1 - 2)	1 (1 - 2)	2 (1 - 3)	2 (1 - 3)	1 (1 - 2)	**<0,001**
If s/he could choose, my child would eat most of the time.	1 (1 - 2)	1 (1 - 2)	2 (1 - 3)	1 (1 - 2)	1 (1 - 2)	**0,04**
If s/he could, my child would always have food in her/his mouth.	1 (1 - 2)	1 (1 - 2)	1 (1 - 2)	2 (1 - 2)	1 (1 - 2)	0,10
My child eats more when s/he is worried.	1 (1 - 2)	1 (1 - 2)	1 (1 - 1)	1 (1 - 2)	1 (1 - 2)	0,67
My child eats more when s/he is annoyed.	1 (1 - 2)	1 (1 - 2)	1 (1 - 2)	1 (1 - 2)	1 (1 - 2)	0,96
My child eats more when s/he is anxious.	1 (1 - 2)	1 (1 - 2)	1 (1 - 2)	1 (1 - 2)	1 (1 - 2)	0,55
My child eats more when s/he has nothing else to do.	2 (1 - 2)	1 (1 - 2)	1 (1 - 2,5)	2 (1 - 3)	1 (1 - 2)	0,71
My child eats less when s/he is angry.	3 (2 - 4)	3 (2 - 3)	2 (1 - 3)	3 (1 - 3)	3 (1 - 3)	0,11
My child eats less when s/he is tired.	3 (2 - 4)	3 (2 - 4)	3 (2 - 4)	3 (3 - 4)	3 (2 - 4)	0,90
My child eats more when s/he is happy.	3 (2 - 4)	3 (2 - 3)	2 (1 - 3)	2 (1 - 3)	3 (1 - 3)	**<0,001**

My child eats less when s/he is upset.	2 (1 - 3)	2 (1 - 3)	2 (1 - 3)	2 (1 - 2)	2 (1 - 3)	0,18
My child finishes their meal fast.	3 (3 - 4)	3 (3 - 4)	3 (3 - 4)	3 (2 - 3)	3 (3 - 4)	**0,02**
My child eats slowly.	4 (3 - 4)	4 (3 - 4)	3 (3 - 4)	3 (2 - 4)	4 (3 - 4)	0,11
My child takes over 30 min to finish a meal.	3 (1 - 3)	2 (2 - 3)	2 (1 - 3)	2 (1 - 2)	2 (1 - 3)	**0,02**
As the meal goes on, my child eats slower.	2 (2 - 3)	2 (2 - 3)	2 (1 - 3)	2 (1 - 3)	2 (2 - 3)	**0,04**
My child loves food.	4 (3 - 5)	4 (3 - 5)	4 (4 - 5)	4 (4 - 5)	4 (4 - 5)	0,06
My child is interested in food.	4 (3 - 4)	4 (3 - 4)	4 (3 - 5)	4 (4 - 5)	4 (3 - 4)	0,08
My child looks forward to a meal.	4 (3 - 4)	4 (3 - 4)	4 (3 - 4)	4 (3 - 4,3)	4 (3 - 4)	0,51
My child enjoys eating.	4 (3 - 4)	4 (3 - 4)	4 (3 - 4)	4 (3 - 4)	4 (3 - 4)	0,55
My child is always asking for a drink.	4 (3,8 - 5)	4 (4 - 5)	4 (3 - 5)	4 (3 - 5)	4 (3 - 5)	>0,99
If allowed, my child would drink constantly throughout the day.	2 (1 - 3)	2 (1 - 3)	2 (1 - 3)	2 (1 - 3)	2 (1 - 3)	0,97
If allowed, my child would always drink something.	2 (2 - 3)	2 (2 - 3)	2 (1 - 3)	2 (1 - 3)	2 (2 - 3)	0,82
My child refuses new food at first.	3 (2 - 3)	3 (2 - 4)	3 (2,5 - 4)	3 (2 - 4)	3 (2 - 4)	0,06
My child enjoys tasting new food.	3 (2 - 4)	3 (2 - 3)	3 (2 - 3)	3 (2 - 4)	3 (2 - 3)	0,24
My child enjoys various foods.	3 (2 - 4)	3 (2 - 4)	3 (2 - 4)	3 (2 - 4)	3 (2 - 4)	0,42

My child is difficult to satisfy with meals.	2 (1 - 3)	2 (1 - 3)	2 (1 - 3)	2 (1 - 3)	2 (1 - 3)	0,13
Even if my child is full, s/he would still consume their favourite food.	2 (1 - 3)	3 (2 - 3)	3 (2 - 4)	3 (2 - 4)	3 (2 - 3)	0,06
My child is interested in tasking food s/he has not tried before.	3 (2,8 - 4)	3 (2 - 4)	3 (2 - 4)	2 (2 - 3)	3 (2 - 4)	0,13
My child decides s/he doesn’t like a food without even trying it.	3 (2 - 3)	3 (2 - 4)	3 (2 - 4)	2 (1 - 3)	3 (2 - 4)	**0,03**
My child has a high appetite.	3 (2 - 4)	3 (2 - 4)	3 (3 - 4)	3 (3 - 4)	3 (2 - 4)	**0,04**
My child leaves food on her/his plate at the end of a meal.	3 (2 - 3)	3 (2 - 3)	3 (2 - 3)	3 (2 - 3)	3 (2 - 3)	0,64
My child gets full before s/he finishes a meal.	3 (2 - 3)	3 (2 - 4)	3 (2 - 3)	3 (2 - 3)	3 (2 - 3)	0,36
My child gets full easily.	3 (3 - 4)	3 (3 - 3)	3 (2 - 3)	3 (2 - 3)	3 (2 - 3)	**<0,001**
My child cannot eat a meal immediately after a snack.	3 (2 - 4)	3 (2 - 3)	2 (1 - 3)	2 (1 - 3)	3 (2 - 3)	**0,03**

*Kruskal Wallis test

Abbreviations: IQR – interquartile range, BM – body mass
