# Supplementary material for: Laying the Groundwork for Health: Eating Behaviour and Physical Activity in Preschoolers in Split-Dalmatia County, Croatia
Source: Children (Basel). 2025 May 29;12(6):699. doi: 10.3390/children12060699 (PMC12191604; doi:10.3390/children12060699)
Supplement: Supplementary file 1 [file children-12-00699-s001.zip › Bucan Nenadic_2025_Children_supplemetary_S3.pdf]

Supplementary material

Table S3. Spearman's correlation indexes of slowness in eating and meal completion.

	**Spearman's correlation index (ϱ) (*p* value)**
	My child eats slowly.
My child leaves food on the plate at the end of the meal.	0.219 (<0.001)
My child gets full before (s)he finishes the meal.	0.142 (0.003)
My child gets full easily.	0.139 (0.004)
